# Nearsighted empathy: exploring the effect of empathy on distance perception, with eye movements as modulators

**DOI:** 10.1038/s41598-024-76731-0

**Published:** 2024-10-24

**Authors:** Soroosh Golbabaei, Khatereh Borhani

**Affiliations:** Institute for Cognitive and Brain Sciences, Shahid Beheshti University, Velenjak, Tehran, Iran

**Keywords:** Empathy, Distance perception, Shared representation, Eye-tracking, Alexithymia, Neuroscience, Psychology

## Abstract

Empathy, a cornerstone of social interaction, involves shared representation, eliciting vicarious emotions. However, its influence on shared perceptual representations, particularly in foundational domains such as distance perception, remains unexplored. In this study, we introduce a novel adaptation of the empathy for pain task to investigate empathy’s influence on distance perception. We also examine how two personality traits, trait empathy and alexithymia, modulate this relationship. Utilizing eye-tracking technology, we examine how attention allocation to different facial and bodily features affects empathy’s impact on distance perception. Our findings indicate that empathy biases individuals to perceive targets as closer, with trait empathy reinforcing this effect and alexithymia attenuating it. Furthermore, we demonstrate that heightened attention to eyes and face correlates with perceiving targets as closer, while attention to hand shows the opposite trend. These results underscore the broader influence of empathy beyond shared emotions, revealing its capacity to alter perceptual processes. By elucidating the interplay between personality traits and visual inputs in shaping these alterations, our study offers valuable insights for future research exploring the role of shared representation in empathy across various perceptual domains.

## Introduction

Empathy serves as a key element in unravelling the complexities in human social interactions. It grants us the ability to share in emotions of others, understand their emotions and adopt perspectives, and care about welfare of others in problem, thereby enabling us to predict their motivations and behaviors and lend a helping hand when needed^1–5^. Despite its recognized importance, there is no general consensus on the definition or the different aspects of empathy. Some theories consider different facets, such as emotion-regulation and the monitoring of inner feelings^6–10^, empathic anger^11–13^, motor empathy^14–16^, affective and cognitive mentalizing^17^, and empathic accuracy or understanding^18^. However, the most commonly used definition of empathy, divides it into two main components: cognitive empathy, which involves the ability to understand and adopt another person’s perspective, and the capacity to infer their mental or emotional state, and affective empathy, which entails sharing of others’ emotions (affect sharing/personal distress) and caring about welfare of others in need (empathic concern)^19–25^. While affect sharing is closely related to personal distress—and the two terms are sometimes used interchangeably in the literature—some argue that they are distinct, with personal distress being self-oriented and affect sharing can be other-oriented^26^. Moreover, while empathic concern often leads to altruistic motivation, high levels of affect sharing might even lead to avoidant behavior^27–33^. Across a plethora of research empathy has been consistently associated with a diverse array of social abilities, including prosocial behavior^4,34–36^, moral development^37,38^, group identification^39^, social competencies^40–42^, and even inhibiting aggressive behavior^43–45^. However, while the influence of empathy on social interactions is well-documented, its intricate interplay with cognition and perception remains a topic of ongoing debate.

A prominent model that connects empathy with cognition is the shared representation model of empathy^46–49^. According to this theory, when we observe or imagine someone experiencing an emotional state, our brain automatically triggers a neural and somatosensory representation of that state within ourselves. This occurs through the activation of mirror neurons, leading to the simulation and formation of a mental representation of the observed emotional state^50–52^. Although this process happens involuntarily and without conscious effort, it can be consciously inhibited or regulated when necessary^51,53–55^. Such shared representations are thought to enhance our perception of others’ emotions^53,56^. Additionally, the extent to which individuals engage in these shared representations varies, influenced by both personal differences and the situational context^10,57^. The general capacity or tendency to empathize with others, often referred to as trait empathy or dispositional empathy, reflects a stable aspect of an individual’s personality. In contrast, the variations in the level of empathy that a person expresses in specific contexts are referred to as state empathy (for a more detailed review see Refs.^24,25,58,59^). Empirical findings have also supported this notion, showing that when we empathize with someone in pain the same neural circuits are activated as if we were feeling the pain ourselves^46,47,60–64^. Furthermore, inducing analgesia not only reduces first-hand pain but also diminishes empathy for the pain of others^65–68^. However, beyond emotions, evidence suggests that shared representation, as induced with enfacement and full-body illusion paradigms, might potentially extend to perceptual domains, blurring boundaries between self and other. Using simultaneous touch to induce shared representation, researchers found that participants felt others’ faces as their own and judged them more similarly^69,70^. This effect was replicated even when participants observed another’s face being touched without being touched themselves^71^ and the effect has shown to be related to heightened dispositional affective empathy (affective empathy measured as a trait)^72^. Moreover, simultaneous tactile stimulation has led to changes in perceived age^73^ and body size^74–76^, self-face processing^77^, altered mood^78^, more accurate depth perception^79^, decreased racial bias^80^, prejudice^81^, and illusionary self-attribution of others’ actions attribution^82^. Thus, if the shared representation model of empathy holds true, such perceptual alterations are likewise expected to manifest during empathic experiences.

Among various perceptual aspects, distance perception stands closely linked to shared representation. It is not merely a rigid, bottom-up process but rather intricately intertwined with self-representation, others’ representations, and individual motivations and goals^83–86^. Previous studies grounded in motivated distance perception^87–89^, have shown that distance perception can be influenced by perceived threat^90^, exclusion^91^, and competition^92^ confirming that perception of distance is linked to socio-emotional experiences. When it comes to interpersonal distance estimation, the reference point is critical. Typically, individuals rely on the self as a primary reference point; thus, any shifts in self-representation can significantly influence the perception of distance^93^. Recent studies have shown that a drift in perceived self-location can occur due to the full-body illusion^94,95^. Therefore, if shared representation is indeed involved in empathic phenomenon, one would expect empathizers to experience a drift in their self-representation toward the target of empathy, resulting in a closer perceived distance. However, despite the potential impact of empathy on distance perception, this relationship remains relatively underexplored, prompting this study to bring this gap to understanding.

On the other hand, both distance perception and empathy require adequate visual information. How individuals allocate their visual attention to the target affects empathy and their representation of the target^96–98^. By comparing visual inputs to past experiences and representations, individuals can simulate a valid representation of others^99^. Altered visual attention to the eyes has been linked to empathy dysfunction in various conditions such as alexithymia^100–102^ and autism spectrum disorder^103–105^. In contrast, increased fixation on the eyes has been associated with trait empathy in some studies^106,107^. Moreover, enhancing empathy through related hormones is demonstrated to increase fixation on faces, compared to other body parts^108^. More specifically, if state empathy influences distance perception, it stands to reason that personality traits influencing state empathy would likewise affect this correlation. Among these personality traits, alexithymia holds particular significance. Alexithymia, characterized by difficulty in identifying and describing emotions, exhibits a negative correlation with trait empathy^101,109–113^. Numerous studies have indicated that individuals with high levels of alexithymia encounter challenges in visually scanning and allocating attention to emotional stimuli, such as the eyes^101,102,114–116^. However, to date, there is no evidence concerning the relationship between gaze behavior during empathy and the degree to which shared representation occur and alters distance perception. Furthermore, the impact of related personality traits such as alexithymia and trait empathy on this relationship remains unexplored.

In the present study, our primary objective was to investigate the impact of empathy on perceived distance between self and others, as well as the influence of eye gaze patterns on such perceptions. To this end, we utilized a modification of the empathy for pain task, during which participants watched images depicting others (targets) at different distances receiving an electrical shock in two conditions of high and low empathy while their eye gaze was recorded. The concept of using high empathy versus low empathy conditions is rooted in previous literature that utilizes these conditions to manipulate empathy^117–122^. This approach offers several advantages over presenting a target in a neutral condition versus a target in pain. Firstly, since we are using eye-tracking to study eye gaze patterns, employing two different pictures could introduce significant differences unrelated to empathy but rather to the pictures themselves (e.g., low level visual perceptual differences). More importantly, it has been demonstrated that this kind of manipulation influences both cognitive and affective empathy, and their effectiveness is not merely a result of experimental cognitive demand^123,124^. Therefore, utilizing the high versus low empathy condition provides the most suitable design for our study. Participants were asked to report their cognitive and affective empathy (the affect sharing/personal distress component) towards each target, as well as estimating the perceived distance to the target. Additionally, we assessed participants’ levels of alexithymia and trait empathy, recognizing their potential modulation of dispositional empathic responses towards others.

We hypothesize that participants will perceive the targets as closer when empathizing with them. Furthermore, we anticipate a correlation between the disparity in cognitive/affective empathy levels between the high and low empathy conditions, and the variance in perceived distance between these two conditions. Previous studies have demonstrated that shared representation and action-simulation mechanisms are more closely associated with affective empathy than with cognitive empathy^125^. Consequently, we anticipate a stronger relationship between affective empathy and distance perception compared to cognitive empathy. However, despite the distinction between these two forms of empathy, they often influence each other^26,126,127^. Additionally, the empathy for pain task used in this study (see Empathy for Pain and Distance Estimation Task) was not designed to manipulate cognitive and affective empathy independently, as this was beyond the scope of our research. Nonetheless, given that shared representation is more strongly linked to affective empathy, we still expect a more pronounced relationship between affective empathy and changes in distance perception.

Regarding eye gaze patterns, we expect to find a positive correlation between differences in fixation on the eyes and face in two conditions, as well as the perceived distance in those same conditions. Finally, we expect that those with higher trait empathy scores and lower alexithymia scores would exhibit less of a difference in perceived distance between the two empathy conditions.

This study aims to shed light on the intricate relationship between empathy, distance perception, and visual attention, further elucidating the mechanisms underlying empathic experiences.

## Method

### Participants

The sample size was determined using a-priori power analysis with the G-Power program^128^. Based on the within-subject design of the study, a power of 0.95, an alpha level of 0.05, and an effect size of 0.5, a minimum sample size of 54 was required. Data collection involved a larger number of participants to account for the potential equipment failures, participant exclusion due to excessive movements or misunderstanding of the task. A total of 60 individuals, (36 Females) participated in the study, with a mean age of 24.33 (*SD* = 5.83). All participants reported no physiological or neurological disorders, were not taking any medication, and had normal or corrected-to-normal vision. Written informed consent was obtained from them before the start of the experiment, and the ethical committee of Shahid Beheshti University approved the study. The experiment was performed in accordance with the ethical committee guidelines and regulations.

### Procedure

Participants completed the Interpersonal Reactivity Index^129^ and Toronto Alexithymia Scale^130^ via an online platform (Porsline) one day before the experiment. They were then invited to the laboratory to participate in the study. Upon arrival, participants were seated on a comfortable chair to get familiarized with the experimenter and the task setting. The procedure of the experiment was then explained to them and they were asked to sit in front of a 17-inch Dell laptop and place their chin on an adjustable chinrest approximately 65 cm away from the screen. Electrocardiogram (ECG) electrodes were attached to the left wrist and left clavicle, while skin conductance responses (SCR) electrodes were attached to the index and middle fingers. The participants were then seated comfortably until the physiological signals stabilized, after which the experiment began. Following eye-tracking calibration (13 points; See Eye-Tracking Analysis), participants were shown two neutral faces (one male and one female, each lasting one minute), different from the study stimuli, and instructed to freely look at them. These images were presented in counterbalanced order. This part was used to evaluate the resting state ECG and SCR and is beyond the scope of this study. Next, the task instructions were illustrated, followed by the empathy for pain and distance estimation tasks.

### Stimuli

Twenty-Four validated images depicting Iranian male and females experiencing pain were adopted from a previous study^102,131^ that were validated in terms of conveying painful expression. These stimuli featured individuals with painful expressions, receiving electrical shocks to their right hands while seated on a chair. The images were taken from eight different individuals (four females), at three different distances. Using images of different distances is intended to incorporate variation in responses to the distance estimation part of the task (See Empathy for Pain and Distance Estimation Task) and to avoid participants from responding indiscriminately. Therefore, participants were required to estimate the target’s distance in each trial, independent of other trials (see^102,131^; for the complete description on preparing and validating the stimuli). Informed consent was obtained for the image presented in this article, allowing its publication in an online open-access journal.

### Empathy for pain and distance estimation task

**Fig. 1:**
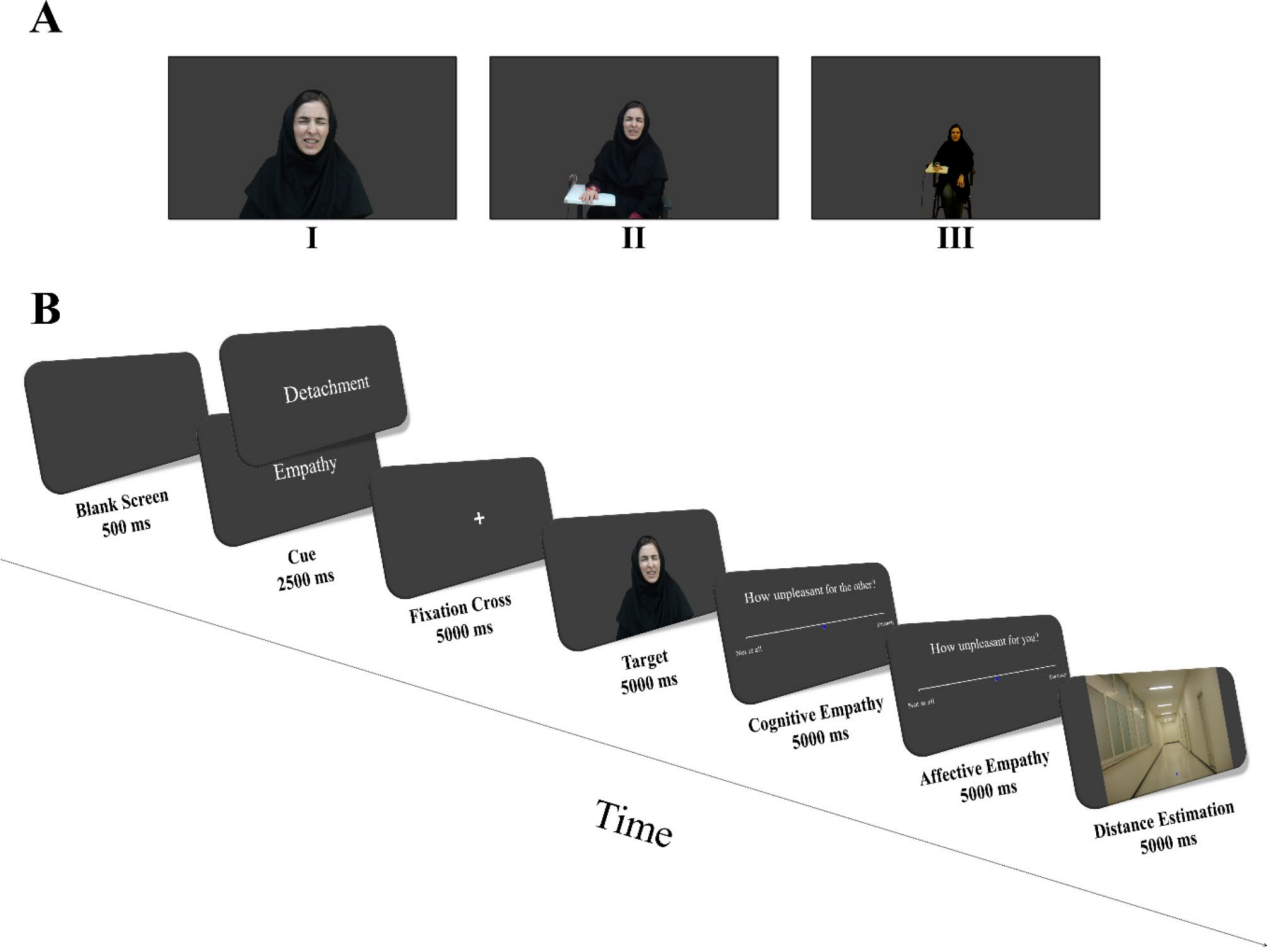
Illustration of empathy for pain task flow and stimuli. (**A**) One of the stimuli depicted at three different distances; (**B**) Graphical representation of the empathy for pain and distance estimation task.

Each trial began with a blank screen for 500 ms, followed by the words Empathy (High Empathy Condition) or Detachment (Low Empathy Condition) for 2500 ms. This indicated whether the participants must empathize with or remain objective and detached towards the person in the following image. Next, a black fixation was shown for 5000 ms in the center of the screen, followed by an image of 5000 ms. Similar to previous studies^132–137^, in response to each image, participants were asked two questions related to empathy: (1) How unpleasant was the stimulus to the other person (Cognitive empathy) and (2) How unpleasant it was to see the other person receive the stimulus (Affect sharing/personal distress component of affective empathy). The questions were answered on a 7-point Likert scale (1: Not at all; 7: Extremely). Answers were not time limited, however, a fixation with the duration of five seconds minus the response time was presented if the participant responded in less than five seconds. Finally, the distance estimation part was presented. The participant was shown with a picture of a corridor with a positionable arrow on it. Using the “up” and “down” keys, they were asked to move and place the arrow where they imagined the person was seated in the previous image. The graphical representation of the trial structure in the experimental task is depicted in Fig. 1.

The participants were presented with images of eight people (4 females), each positioned at three different distances, resulting in 24 unique photographs (8 × 3 = 24). Additionally, each unique image was presented twice, once when empathizing (High Empathy Condition), and once when detaching (Low Empathy Condition). Thus, there were 48 trials in total in the Empathy for pain and distance estimation task. Pseudo-randomization was used to avoid displaying two pictures of the same person in a subsequent order. The task was created and presented in Psychopy.

Responses to cognitive empathy, affective empathy, and distance estimation were averaged across trials of high and low empathy conditions. Moreover, the difference between two conditions (High empathy – Low empathy) were calculated and used in subsequent analyses (see statistical analysis).

### Eye-tracking analysis

An SMI Red-250 was used to record eye movements with a sampling frequency of 250 Hz. A 13-point calibration and verification procedure were used and repeated if deviations on X or Y axis were above 0.5 degree. Begaze software was used to analyze eye-movement recordings. Three participants with tracking ratios below 80%^138–141^ were excluded prior to analysis, leaving 58 participants in the eye-tracking analysis. Areas of interest (AOIs) were next defined as the eyes, face, and the hand that received the shock. As in previous studies^106,142,143^, a rectangle was used for the eyes AOI, vertically expanded from the top of the eyebrows to an equivalent distance below the eyes and horizontally extended to the side of the face.

### Self-report questionnaires

#### Interpersonal reactivity index (IRI)

Empathy was measured using the validated 16-item Farsi version of IRI^109^, comprising four dimensions of empathic concern, perspective taking, personal distress, and fantasy. Each item is responded to on a 5-point Likert scale (0 = Does not describe me well; 4 = Describes me well). Good internal consistency (in the range of 0.67 − 0.71) and test-retest reliability (in the range of 0.71 − 0.84) is reported for the scale.

#### Toronto alexithymia scale (TAS)

As a proxy for difficulty in identifying and describing emotions, the Alexithymia score was measured using the validated Farsi version of TAS^144^. TAS is a 20-item questionnaire, measuring alexithymic aspects in three subscales of difficulty in identifying emotions (7 items), difficulty in describing emotions (5 items), and externally oriented thinking (8 items), where each item is rated on a 5-point Likert scale (1 = completely disagree; 5 = completely agree). Internal consistency and test-retest reliability are reported to be in the range of 0.72 to 0.82 and 0.80 to 0.85 respectively.

### Statistical analysis

Paired sample t-test was used to compare distance estimation, as well as cognitive and affective empathy under two conditions of high and low empathy. We next calculated the difference between these three measures under two conditions (e.g., distance estimation at low empathy condition subtracted from distance estimation at high empathy condition; from now on presented as Distance Estimation_Diff_), and calculated the correlation between them. Furthermore, we examined how alexithymia and trait empathy scores correlate with differences in distance estimation. These relationships were investigated using Pearson correlation. Finally, using the same procedure, we calculated the difference between eye-movement measure under two conditions, and explored the correlation between gaze-pattern and distance estimation.

It is worth noting that a negative value in cognitive empathy_Diff_ and affective empathy_Diff_ indicates greater empathy towards the target in the high empathy condition versus the low empathy condition (same for the gaze-pattern measures). On the other hand, lower values in distance estimation suggest a closer estimation. Therefore, negative values in distance estimation_Diff_ indicate that participants perceived the distance as closer in the high empathy condition compared to the low empathy condition.

## Results

### Manipulation check

**Fig. 2:**
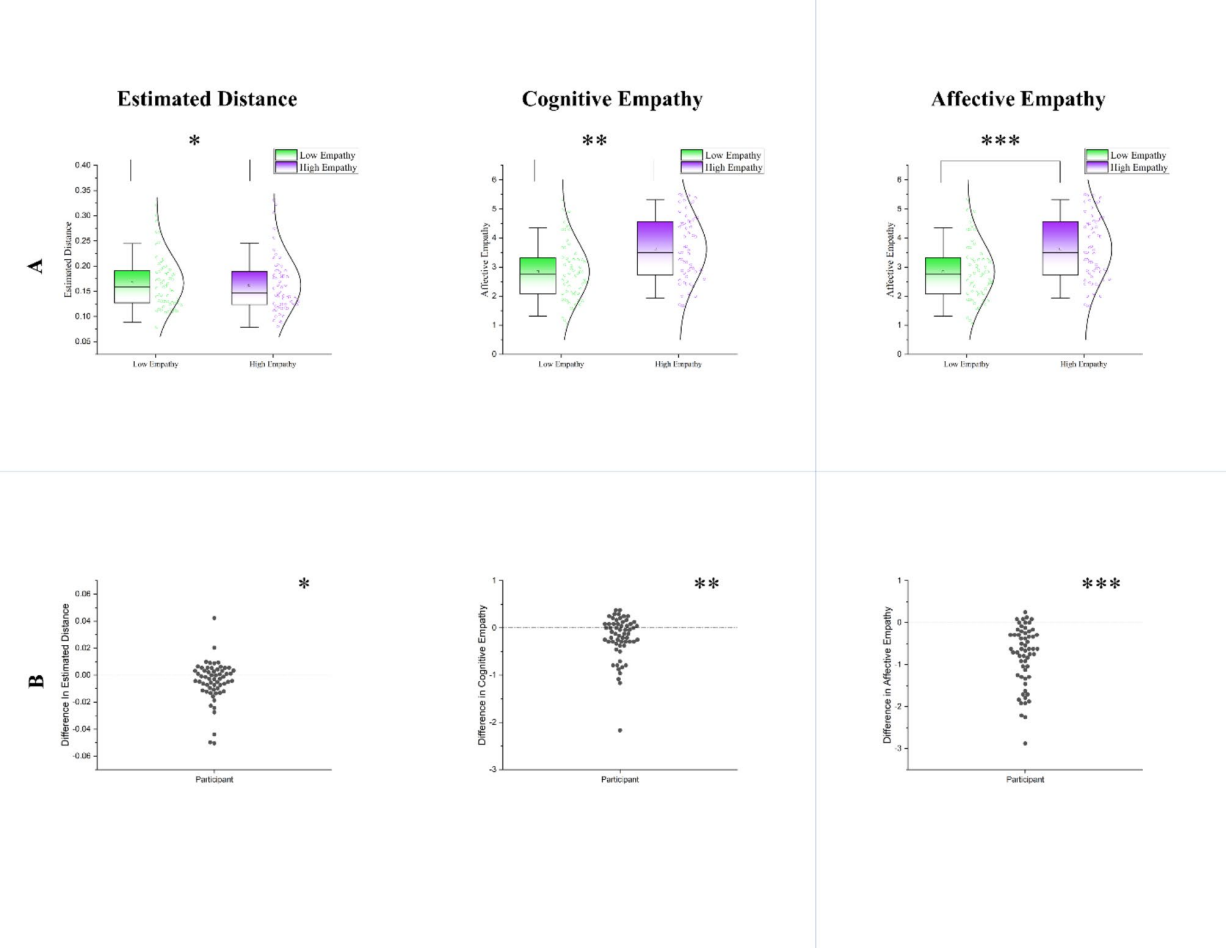
Comparison of distance estimation, cognitive empathy, and affective empathy across high and low empathy conditions. (**A**) Distribution of each measure across two condition; (**B**) Distribution of difference in each measure (high empathy – low empathy); Higher scores in difference cognitive empathy and difference in affective empathy means that participants have reported higher cognitive and affective empathy in high empathy condition compared to low empathy condition respectively; Whereas, higher values in Difference in Distance Estimation indicate further estimation and lower values indicate closer estimation of distance in high empathy condition compared to low empathy (Lower scores in distance estimation are indicative of perception of targets as closer). Estimated distances are quantified on a scale from zero to one, with zero denoting the lower edge and one indicating the upper edge of the screen. Lower values in distance estimation indicate closer estimations. *p <  0.05; **p < 0.01; ***p <  0.001.

**Fig. 3:**
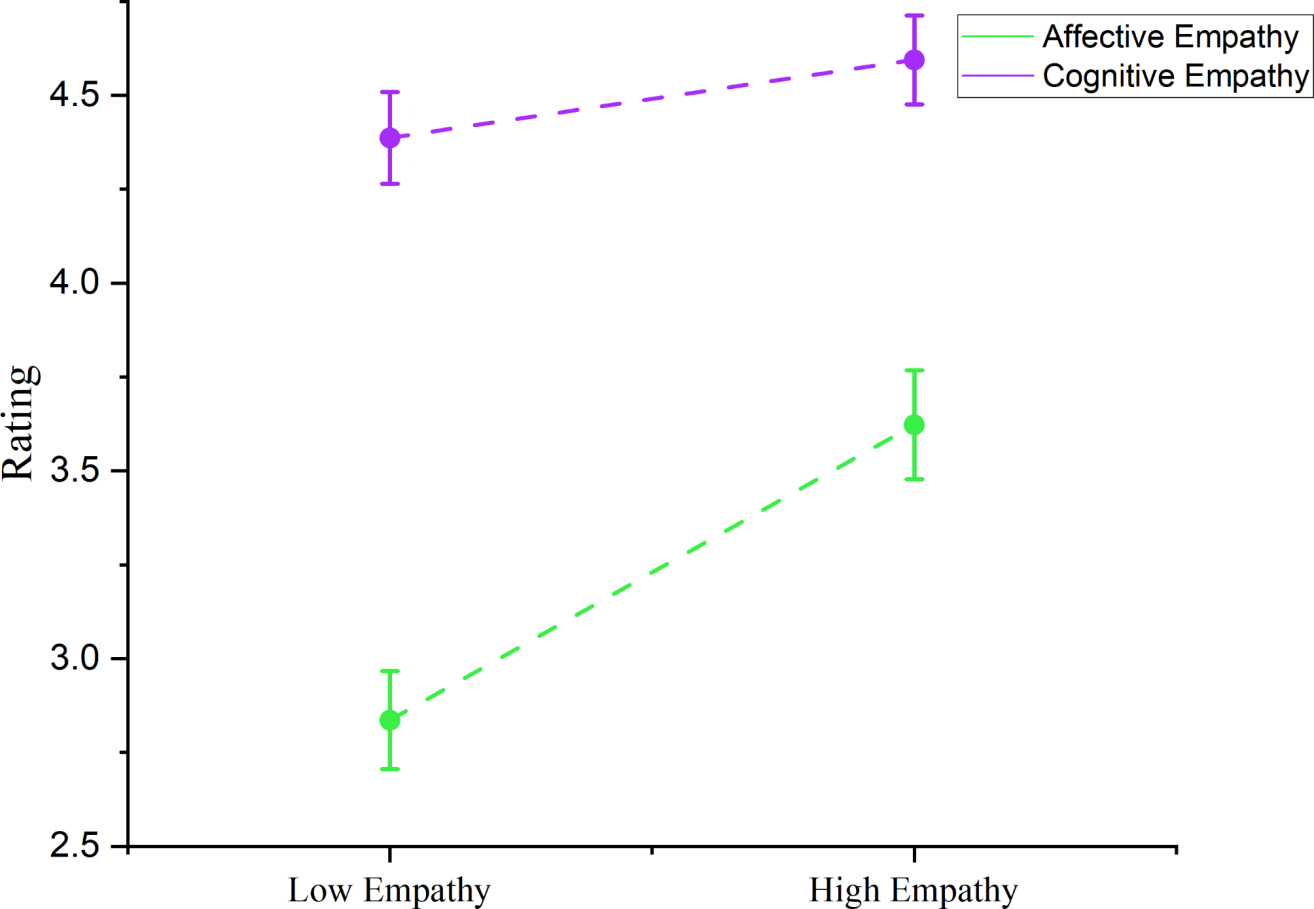
Mean and standard error of cognitive and affective empathy in high and low empathy conditions. Error bars indicate standard error of the mean. The interaction effect of empathy component (Cognitive/Affective) and condition (Low/High Empathy) is significant.

A 2 (cognitive/affective) × 2 (low/high empathy) repeated measures ANOVA, revealed significant main effects of both the empathy component (cognitive/affective), *F*(1, 59) = 176.495, *p* < .001, and the condition (low/high empathy), *F*(1, 59) = 61.403, *p* < .001 (Table 1; Fig. 2). Additionally, there was a significant interaction between the empathy component and condition, *F*(1, 59) = 45.873, *p* < .001 (Fig. 3). To explore these findings further, post-hoc analyses were conducted by comparing cognitive and affective empathy scores between the high and low empathy conditions using two paired sample t-tests. When empathizing with targets, participants reported significantly higher levels of both cognitive empathy, *t*(59) = 3.563, *p* = .001, and affective empathy, *t*(59) = 8.633, *p* < .001 (Table 1; Fig. 2). Furthermore, participants scored higher in cognitive empathy compared to affective empathy in both the low empathy condition, *t*(59) = 14.268, *p* < .001, and the high empathy condition, *t*(59) = 9.785, *p* < .001. However, as illustrated in Fig. 3 and supported by the significant interaction effect, our manipulation had a stronger impact on affective empathy than on cognitive empathy. We also found a significant correlation between cognitive and affective empathy in both low, *r*(59) = 634, *p* < .001, and high empathy conditions, *r*(59) = 735, *p* < .001. Detailed information regarding the distribution of cognitive and affective empathy across conditions is presented in Table 1; Fig. 2.

**Table 1:** Means, standard deviations, and t-test statistics for distance estimation, cognitive, and affective empathy across two conditions.

Variable	High empathy		Low empathy		t	df	*p*	d
	M	SD	M	SD				
Distance estimation	0.162	0.056	0.166	0.052	2.354	59	0.022	0.303
Cognitive empathy	4.594	0.919	4.387	0.951	3.563	59	0.001	0.460
Affective empathy	3.623	1.126	2.837	1.012	8.633	59	< 0.001	1.113

Estimated distances are quantified on a scale from zero to one, with zero denoting the lower edge and one indicating the upper edge of the screen. Lower values in distance estimation indicate closer estimations.

### Distance estimation

**Fig. 4:**
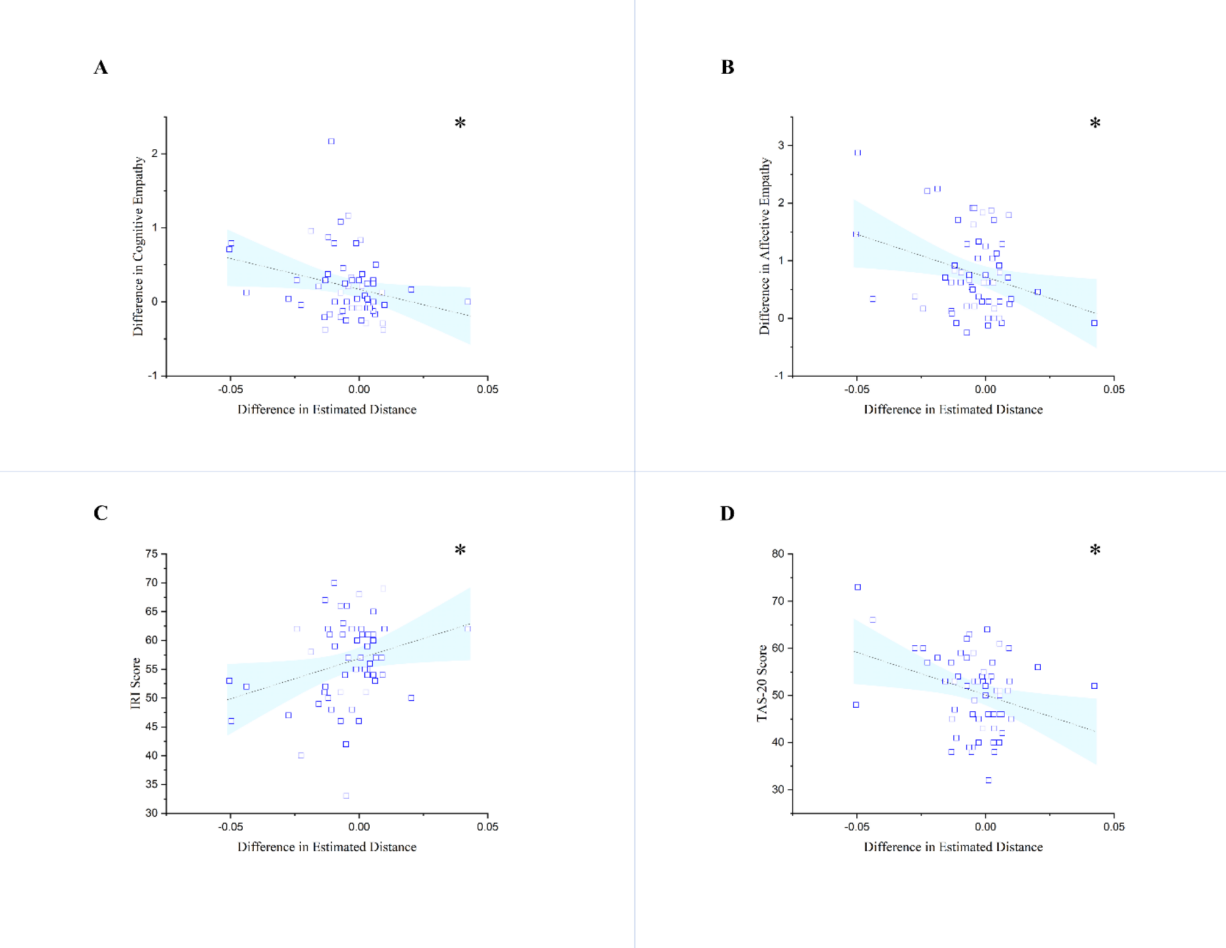
Scatter plots of difference in estimated distance based on difference in cognitive and affective empathy, IRI, and TAS-20. (**A**) Scatter plot and regression line of difference in estimated distance and cognitive empathy; (**B**) Scatter plot and regression line of difference in estimated distance and affective empathy; (**C**) Scatter plot and regression line of difference in estimated distance and Interpersonal Reactivity Index (IRI); (**D**) Scatter plot and regression line of difference in estimated distance and Toronto Alexithymia Scale (TAS-20); Higher scores in difference in cognitive empathy and difference in affective empathy mean that participants have reported higher cognitive and affective empathy in high empathy condition compared to low empathy condition respectively; Higher values in distance estimation indicate further estimation and lower values indicate closer estimation of distance in high empathy condition compared to low empathy. (Lower scores in distance estimation are indicative of perception of targets as closer). *p <  0.05.

Utilizing a paired sample t-test we found that participants significantly estimated the distances closer when empathizing, rather than when detached *t*(59) = -2.354, *p* = .022 (Table 1; Fig. 2). In addition, Distance estimation_Diff_ (i.e., subtracting distance estimated in the high empathy condition from the distance estimated in the low empathy condition) showed a significant negative correlation with both cognitive empathy_Diff_, *r*(59) = − 0.270, *p* = .037, and affective empathy_Diff_, *r*(59) = − 0.308, *p* = .017. Thus, the greater the difference in cognitive empathy (as well as affective empathy) between two conditions (i.e., higher cognitive empathy in high empathy condition, compared to low empathy condition), the closer the participants estimated the distance of targets in high empathy versus the low empathy condition. Next, we examined the relationship between distance estimation_Diff_, trait empathy, and alexithymia. Distance estimation_Diff_ showed a significant positive correlation with IRI, *r*(59) = 0.279, *p* = .031, and a significant negative correlation with TAS-20 scores, *r*(59) = 0.319, *p* = .013. Detailed results are presented in Table 2; Fig. 4.

**Table 2:** Intercorrelations for study variables.

Variable	1	2	3	4	5
1. Distance estimation_Diff_	–	− 0 0.270*	− 0.308*	0.279*	− 0.319*
2. Cognitive empathy_Diff_		–	0.414**	− 0.121	0.126
3. Affective empathy_Diff_			–	− 0.330*	0.176
4. IRI				–	− 0.080
5. TAS					–

Distance estimation_Diff_: The difference between the estimated distances in the high empathy condition and those in the low empathy condition (High empathy condition – Low empathy condition); Cognitive empathy_Diff_: The difference between the cognitive empathy in the high empathy condition and those in the low empathy condition (High empathy condition – Low empathy condition); Affective empathy_Diff_: The difference between the affective in the high empathy condition and those in the low empathy condition (High empathy condition – Low empathy condition); Higher values in distance estimation indicate further estimation and lower values indicate closer estimation of distance in high empathy condition compared to low empathy. *p < .05; **p < .01.

### Eye-tracking analyses

**Fig. 5:**
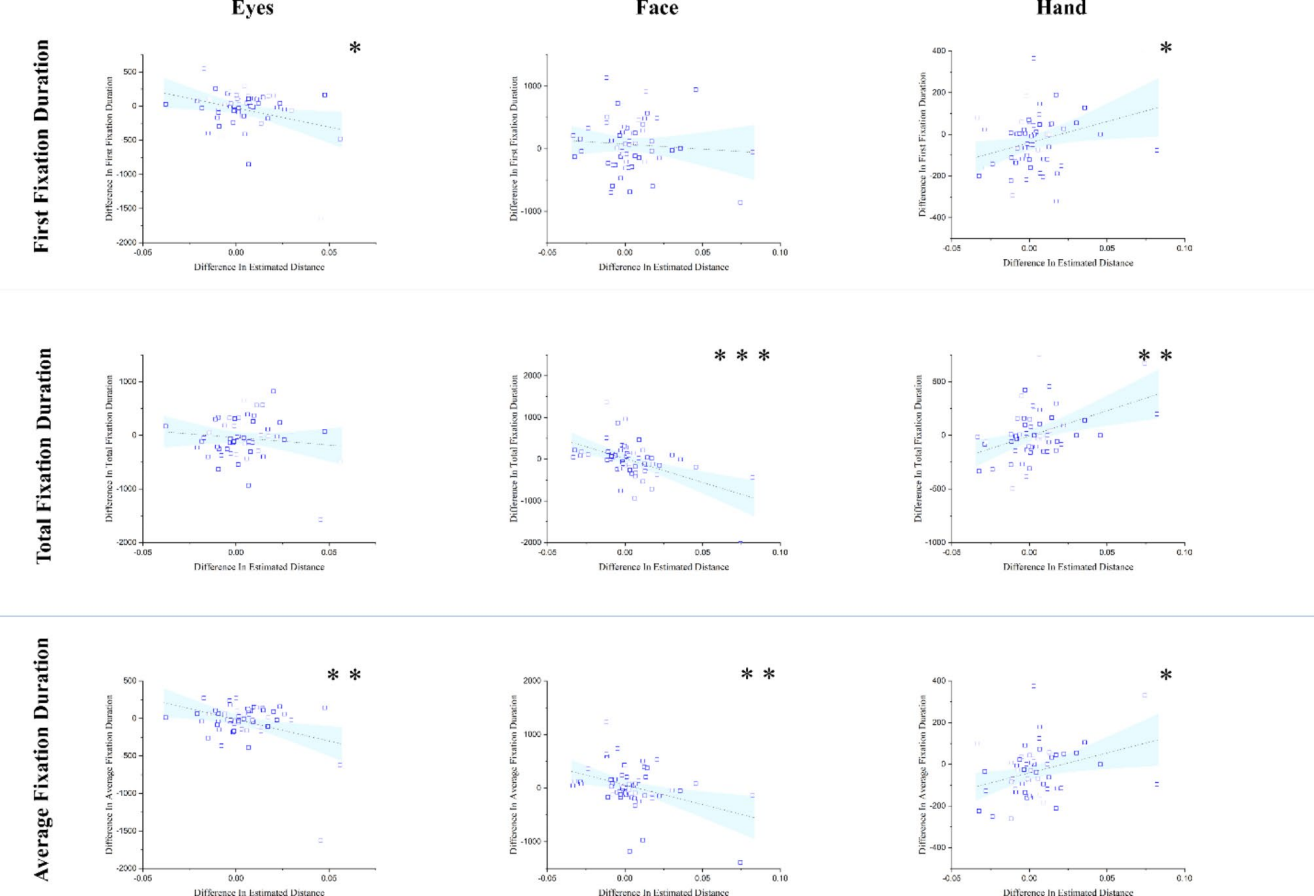
Scatter plots of difference in estimated distance based on eye-movement measures in different areas of interests. Higher values in distance estimation indicate further estimation and lower values indicate closer estimation of distance in high empathy condition compared to low empathy. Differences in eye-movement measures are calculated by the subtraction of eye-movement measures in low empathy condition from the high empathy condition (e.g., difference in first fixation duration = first fixation duration in high empathy condition – first fixation duration in low empathy condition); *p <  0.05; **p <  0.01; ***p <  0.001.

First, we examined the correlation between distance estimation_Diff_ and three measures of eye movement, namely total fixation duration_Diff_, average fixation duration_Diff_, and first fixation durationDiff. In the eyes AOI, distance estimation_Diff_ was negatively correlated with first fixation durationDiff, *r*(57) = − 0.301, *p* = .023, and average fixation duration, *r*(57) = − 0.354, *p* = .007, but not total fixation duration, *r*(57) = − 0.117, *p* = .386. In other words, the longer the first fixation duration and the longer the average fixation duration in the eyes region, the closer the distance estimation was. Moreover, distance estimation_Diff_ was negatively correlated with both total fixation duration, *r*(57) = − 0.507, *p* < .001, and average fixation duration, *r*(57) = − 0.375, *p* = .004, in the face AOI and positively correlated with the total fixation time, *r*(57) = 0.414, *p* = .001, the first fixation time, *r*(57) = 0.306, *p* = .021, and with the average fixation time, r(57) = 0.320, *p* = .015 in the hand AOI (Table 3; Fig. 5).

**Table 3:** Correlation between difference in estimated distance and eye-movements measures in different areas of interests.

Variable	Eyes	Face	Hand
First fixation duration_Diff_	− 0.301*	− 0.080	306*
Total fixation duration_Diff_	− 0.117	− 0.507***	0.414**
Average fixation duration_Diff_	− 0.354**	− 0.375**	320*

First fixation duration_Diff_: The difference between the first fixation duration in the high empathy condition and those in the low empathy condition; Total fixation duration_Diff_: The difference between the first fixation duration in the high empathy condition and those in the low empathy condition; Average fixation duration_Diff_: The difference between the first fixation duration in the high empathy condition and those in the low empathy condition. *p < .05; **p < .01; ***p < .001.

## Discussion

Empathy, a nuanced phenomenon extending beyond shared emotions, has been an intriguing subject in studies investigating its impact on perception and cognition. Nevertheless, the impact of empathy on distance perception remains unexplored. To delve deeper, the question arises: does engaging in empathetic experiences, adopting others’ perspectives, and putting ourselves in their shoes, lead to a transformative shift in our real-life perception of distance? This study sought to fill this gap by employing a modified empathy for pain task, examining how empathy influences not only emotional experiences but also alters our perception of physical distance. Additionally, we investigated the interplay of eye gaze patterns, trait empathy, and alexithymia in shaping this intriguing connection.

Our results uncovered a compelling insight: under high empathy condition, participants consistently perceived the target as physically closer. This not only aligns with an established theory such as shared representation^46–49^, but also extends our understanding of empathy by revealing that empathy goes beyond understanding and sharing emotions of others and caring about them. While previous research has primarily focused on the emotional effects of shared representation and empathy, our study highlights that these processes may also influence perceptual experiences, such as the perception of distance.

The observed phenomenon may partially stem from the concept of shared representation and simulation. As participants engage in empathetic experiences, mirror neurons fire, mimicking the feelings of the target of empathy^145–148^. Some theories suggest that this neural activity prompts us to simulate the sensorimotor, affective, and mental states of the target, effectively allowing us to project ourselves into the other person’s perspective^10,50,52,149^. If such a simulation occurs during empathy, it could also influence the perception of emotional proximity or distance between the empathizer and the target.

Alternative explanations also exist. Theories such as motivated distance perception^87–89^ and the new look perspective^150–152^ propose that desires, motivations and goals can significantly influence our perceptions. While the impact of motivation has not been investigated in the context of empathy, evidence from related studies suggest its significance. For instance, studies reveal that thirsty individuals tend to perceive a bottle of water as closer^88^, and individuals often view desired locations as more vivid and proximate^153^. Competitive contexts may contribute to a perception of greater distance between individuals, in contrast to cooperative situations^154^. When empathizing with others, individuals are motivated to alleviate the suffering of those around them^5,155^. This motivation may indeed influence how we perceive the distance to that person, highlighting the intricate interplay between cognitive processes and emotional motivations.

Furthermore, we also found a correlation between both affective and cognitive state empathy and altered distance estimation, where those with higher levels of empathy estimated the distances as closer. This robust confirmation, independent of instructional variations in high and low empathy conditions, underscores the consistent impact of the level of empathizing on distance perception. Moreover, while some previous studies have discerned differences between cognitive and affective empathy in various psychological phenomena^29,156–162^, with shared representation being more closely related to affective empathy, we anticipated a stronger correlation between changes in affective empathy and changes in distance estimation compared to cognitive empathy. However, our findings revealed only a subtle difference between the two, suggesting that the relationship between empathy types and distance estimation may be more complex than initially expected. Thus, our results indicate a convergence of these components in the realm of distance perception, where they are both of the same importance. To scrutinize this convergence, it’s essential to consider the interconnected nature of affective and cognitive empathy^23^. Affect sharing/personal distress aspect of affective empathy involves sharing the emotional experiences of others, while cognitive empathy involves understanding their mental and emotional states^24^. The convergence in altered distance perception suggests a holistic engagement with empathetic processes, wherein both understanding and sharing play integral roles. This aligns with recent theories^163–166^ highlighting the intertwined nature of affective and cognitive empathy, which, despite being distinct, often interact in most common situations^26,126,127^. Yet, it is noteworthy that we did not manipulate these components separately, and our results are based on the participants’ reports of their own cognitive and affective empathy. Thus, the similarity found between these components should be treated with caution.

We also examined the effect of two personal characteristics, namely alexithymia and trait empathy on distance perception. As expected, alexithymia displayed a negative correlation with the perceived distance difference between conditions, while trait empathy exhibited a positive relationship. While at first glance alexithymia is expected to be negatively correlated to state empathy and thus positively affect distance perception (and vice versa for trait empathy), we did not design this study to evaluate state empathy and distance perception in isolation (i.e., a single condition), but focused on the disparity between two conditions. In order to satisfy the task needs, participants had to adapt their emotions and reactions, so that they would empathize in one condition and not in the other one. Thus, those with lower levels of trait empathy and emotional contagion are expected to show lower levels of empathy in the low-empathy condition, resulting in higher difference between two conditions. Alexithymia, characterized by difficulties in identifying and describing one’s own emotions, may impact the ability to resonate with the emotional experiences of others^102,110–112,116,167,168^. This, in turn, could result in a reduced affective empathy^55,169–171^, leading to a more pronounced difference in perceived distance between two conditions. In conclusion, these findings align with the central theme of this study, demonstrating that alterations in distance perception are not only influenced by state empathy but also by individual traits associated with empathy.

Finally, our exploration into the role of gaze patterns on perceived distance provided intriguing revelations. Participants with longer first fixation and average fixation duration on the eye regions during the high empathy condition, perceived the targets as closer. This aligns with prior studies highlighting the significance of eye-contact in empathy. Several studies have shown that trait empathy is related to more looking to the eyes^106,107,172,173^. Eye region is recognized as the most affective facial region, conveying a heightened level of emotional inputs compared to other facial regions^106,174^. Specifically, it is widely acknowledged that eye contact plays a pivotal role in human communication, fostering connection and mutual understanding^175–177^. Further, its significance extends to the promotion of synchronization and emotional contagion^106,178,179^. However, our study suggests that this fundamental element goes beyond mere communication, influencing the very perception of distances between individuals. This interplay between nonverbal cues and perceptual alterations underscores the intricate ways in which our perceptual experiences are intertwined with social and emotional processes.

This finding can also be linked to both shared representation and motivated distance perception. In the context of shared representation, heightened attention to the eyes signifies an increased input of visual pain, resulting in a more profound shared representation and subsequently, an altered perception of distance. On the other hand, within the framework of motivated empathy, increased eye contact may invoke a deeper understanding of others’ pain, resulting in heightened motivation to help and greater inclination to perceive others as closer. Moreover, our results regarding the gaze pattern extended beyond the eyes to encompass differences between looking at the face and the body. Those who gazed more at faces and less at hands, were inclined to perceive the target as closer in the high empathy condition compared to the low empathy condition. This discrimination in gaze between the face and the body has also been documented previously, where those with higher trait empathy were found to look at the faces as a more socially informative compared to the body. Despite hands being the target of the delivered pain in our study, it appears that, irrespective of the shock received, faces played a more pivotal role in the empathic phenomenon and influenced changes in perceived distance. However, it is important to note that, due to the static nature of images, less pain information was conveyed through hands than faces. As a result, this finding warrants cautious generalization and is in need of further investigation.

Despite the promising results, our study has some limitations. The exploration of empathy, being inherently interpersonal, calls for future studies adopting dyadic designs to unravel the intricacies of the interaction between empathizer and target of empathy. Dyadic designs offer a more ecological representation of empathetic processes, allowing researchers to explore how the reciprocal interactions between individuals influence not only emotional experiences but also perceptual phenomena. However, in this study, we employed a single-subject design to mitigate the confounding effects inherent in dyadic experiments. This approach provided greater control over potential variables and created an optimized environment for the examination of eye gaze patterns. Moreover, the static nature of our images limits the dynamic aspects of painful postures and gestures, urging subsequent investigations to employ dynamic stimuli for a more comprehensive understanding. Incorporating dynamic stimuli in future studies can capture the fluidity and complexity of empathetic experiences, providing a more realistic portrayal of how our perceptions evolve in real-time social interactions. Furthermore, our assessment of cognitive and affective empathy relied on participants’ responses. Given the complex nature of empathy, there is a possibility that participants may respond to cognitive empathy questions based on their own affective responses, which could result in a high correlation between cognitive and affective empathy scores. Alternative tasks such as the multifaceted empathy task (MET^180^) employ categorical responses for different emotions in the cognitive empathy question, requiring participants to identify the correct emotion of the target. However, since our study focused on pain, this option was not applicable. Furthermore, our study focused exclusively on the affect sharing/personal distress aspect of affective empathy. Given that previous research has primarily highlighted differences between empathic concern and personal distress^11,27,33,155^, future studies could benefit from incorporating assessments of both components to better understand their potential differential effects on distance perception. For example, some studies have questioned the degree to which a participant would be willing to help another person if given the opportunity, as a way to assess empathic concern^181^. Although, the task employed in our study is a well-established and widely-used measure, future studies may explore alternative designs to independently manipulate cognitive and affective empathy, thereby offering a more nuanced exploration of their respective effects.

To summarize the present study evaluated the impact of empathy on distance estimation. Our results indicate that individuals, when immersed in empathetic experiences, perceive targets as closer, with both cognitive and affective empathy playing a crucial role in this perceptual alteration. This promising finding underscores that empathy not only influences our emotional representations but also induces changes in our perceptual representations. While our study was focused on distance perception, it opens a new area of research prompting exploration into the potential extent of shared representation. Furthermore, as a confirmation of our findings, we showed that personality characteristics such as alexithymia and trait empathy affect the degree to which distance perception is altered in empathetic situations. Finally, we illustrated that gaze pattern significantly contribute to the alteration of perceived distance in empathy. Specifically, we showed that heightened fixation on the target’s eyes corresponds to a closer perceived distance. Additionally, compared to the body, face holds greater importance in shaping this altered perception. These nuanced insights contribute to a deeper understanding of the intricate interplay between empathy, individual characteristics, and visual attention, opening avenues for future investigations into the dynamics of social cognition.

## Data Availability

The data that support the findings of this study are available upon reasonable request from the corresponding author.
